# Fatigue Property of Open-Hole Steel Plates Influenced by Bolted Clamp-up and Hole Fabrication Methods

**DOI:** 10.3390/ma9080698

**Published:** 2016-08-16

**Authors:** Zhi-Yu Wang, Lihui Li, Yong-Jie Liu, Qing-Yuan Wang

**Affiliations:** 1 Department of Civil Engineering & Mechanics, Sichuan University, Chengdu 610065, China; zywang@scu.edu.cn (Z.-Y.W.); 18200340772@163.com (L.L.); liuyongjie@scu.edu.cn (Y.-J.L.); 2 Sichuan Provincial Key Laboratory of Failure Mechanics and Engineering Disaster Prevention & Mitigation, Sichuan University, Chengdu 610065, China; 3 School of Architecture and Civil Engineering, Chengdu University, Chengdu 610106, China

**Keywords:** open-hole, bolted clamp-up, fatigue property, life assessment

## Abstract

Steel plates with open holes are commonly used in structural assemblies. The fatigue properties of such details are influenced by bolted clamp-up and hole fabrication methods. The fracture surface, stiffness degradation and fatigue life of test specimens are investigated in detail and compared with the contemporary test data. The analysis results show that the presence of draglines greatly influences the fatigue crack initiation at the open-hole cut by laser. The bolted clamp-up condition greatly enhances the stiffness and the fatigue life of the open-hole details. A discussion is also made from a comparison with the referred fatigue life of hole fabrication details, such as the influence of plate thickness and plasma cutting, drilling and oxy-fuel gas cutting, with the details studied herein. This work could enhance the understanding of the fatigue property and design of such details.

## 1. Introduction

Steel plates with open holes are commonly used in structural components to enable an assembly with bolts. The gusset plates and cross-frames are components which can be regarded as examples of such types. As evidenced from the basic configurations of these components, the weaknesses of the components are determined by fabrication methods as well as clamping conditions [1,2], which become an important issue for fatigue loading cases.

For the sake of evaluating the mechanical characteristics of the components with hole fabrication, tensile tests have been widely used in obtaining the basic mechanical properties of the connections. Punched holes as a fast and cost-effective method of forming holes are widely used for secondary tension members. Also, holes may be formed by full-size drilling and thermal cutting, which a concern of most early studies. From the tests, Chesson and Munse [3] concluded that punching reduced the ductility of the net section which causes the early arrival of ultimate stress near the holes and thus limits the effectiveness in strength development. Frank [4] showed that the strength ratio of drilled-hole specimens was much lower than that of punched-hole specimens and pure punching was recommended in secondary connection members. Later, Rassati et al. [5] confirmed this point and observed a decrease in the tensile ductility for punched-hole specimens. In agreement with all these findings in the literature, the AASHTO LRFD (American Association of State Highway and Transportation Officials Load-and-resistance Factor Design) [6] restricts pure punched holes in the primary loading carrying members and gives limits of material thickness in some other uses. In addition, a considerable amount of recent research [7,8,9,10,11] was developed based on the open-hole tension and bolt-filled hole tension tests in accordance with ASTM (American Society of Testing Materials) standards [12] to provide mechanical characteristics of composite laminates.

Although the weakness of punched holes may be improved using reamed full-size to remove the damaged zone surrounding the hole, laser cutting as a substitute has shown good applications in engineering [13,14]. The merits of laser cutting are obvious, including the exemption from physical contact and mechanical force, low-cost fast processing and precise operation without changing tools. Yilbas et al. [15] showed that the small holes made by laser cutting in aluminium foam are parallel sided and free from defects such as sideways burning and large burr formation. Alegre et al. [16] concluded from the fatigue tests that the punched-hole specimens have a much lower life span than replicated drilled-hole ones and they have fatigue cracks initiated at the transition point. Sánchez et al. [17] observed that the drilled specimens have twice the fatigue resistance of the punched specimens. Brown et al. [18] confirmed the conclusion from [16,17] and showed that specimens with laser-cut holes have better surface conditions but the same average strength when compared with punched-hole specimens. Garcia et al. [19] and Cicero et al. [20] reported research comparing the fatigue performance of drilled and punched holes with typical thermal cutting methods. Due to limited studies, however, a full understanding of the fatigue property of open-hole steel plates when the modern fabrication process and clamping conditions are concerned has not yet been developed.

In this paper, the fatigue properties of open-hole steel plates using laser cutting as a modern and common fabrication method will be studied in terms of failure mode and life results. Also, the fatigue life of the open-hole steel plates with the bolted washer clamp-up condition is also examined. Finally, the effects of clamping and fabrication methods on the fatigue life of such structural details will be discussed further. The results obtained in this study can be taken as a basic reference for further study involved in the fatigue life extension of related structural details.

## 2. Material and Fatigue Test Procedures

The material used for the test specimens was Grade 345B low alloy steel which conforms to the Chinese national standard GB/T1591. This material has good toughness and ductility. The chemical composition and mechanical properties are listed in Table 1. Related information on steel materials of S355N, S460Q and S460M in [17,19] is also given herein as a reference and for discussion in the subsequent section.

**Figure 1:**
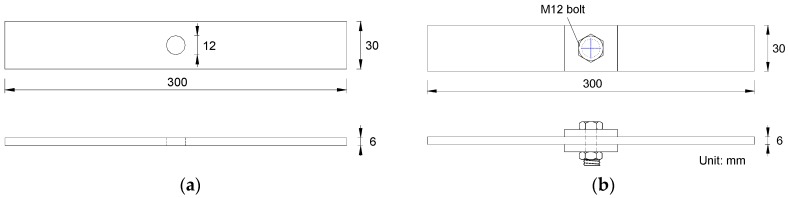
Geometry of typical test specimens. (**a**) Open-hole details; (**b**) Bolted washer clamp-up details.

The test specimens were designed to consider two configuration details, i.e., the open-hole details and the bolted washer clamp-up details. All test specimens had similar dimensions, as shown in Figure 1. The steel plate in tension was 6-mm thick. The plates were machined to their design dimensions of 300 mm long and 30 mm wide prior to the holes being made. The hole at the centre of the plate was cut using a standard laser cutter. During the cutting process, the metal workpiece was completely penetrated by the laser. A stream of gas blew off the melted materials to form a hole as the laser beam moved around the melting material. The metal laser-cutting machine of 2000 W was employed in this cutting process. In this machine, the nozzle diameter of 1.6 mm was chosen to ensure that the gas diffusion area and size can be properly controlled. Meanwhile, the laser cutting was handled with the speed of 1.5 m/min to enable stable cutting. For the test specimens with bolted washer clamp-up details, the square washer placed beneath the bolt head and nut in double-clamping the steel plate was kept at 6 mm thick and 30-mm wide. All washer surfaces were flattened without rust and with a bevelled edge to ensure a good contact condition. The M12 high strength bolt with the tightened torque of 79 N·m was chosen for the bolted connection following the Chinese code GB/T 16823.2-1997 [21] which gives the general rules of tightening for threaded fasteners.

The test specimens were tested using the Shimadzu EHF-UV050k2-020-0A fatigue testing machine of 50 kN capacity. Output and instant information from the testing machine were monitored and recorded by an automatic data acquisition system controlled by Windows Software for 4830 3.40b. Tensile fatigue tests were conducted following the Chinese code GB/T 3075-2008 [22]. Constant amplitude sinusoidal stress cycles with the frequency of 8 Hz were conducted during the fatigue test. Five target stress levels between 200 and 340 MPa were mainly applied. The stress ratio was set at 0.1 for all tests. Fifteen specimens with open-hole details followed by six specimens with bolted washer clamp-ups were tested. The fatigue life was determined as the specimen was tested to rupture. The actual measured cross-sectional dimensions were used for the calculation of the nominal stress of the testing specimens.

## 3. Experimental Test Results

### 3.1. Fracture Surface Observation

**Figure 2:**
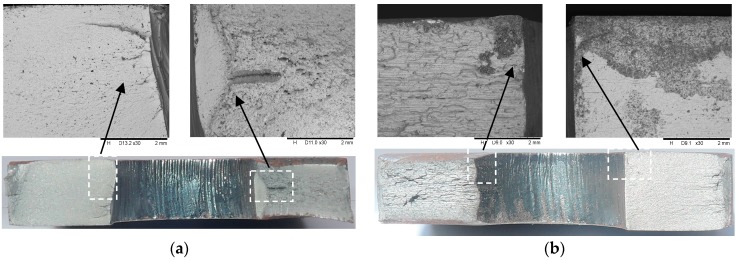
Typical fracture surface of test specimens. (**a**) Without bolted clamp-up (Δσ = 220 MPa); (**b**) With bolted clamp-up (Δσ = 300 MPa).

The fractures of the steel plate exclusively originated at the edge of the hole. The typical crack initiation through the thickness of the plate around the hole appeared to be unsymmetrical on both sides of the hole. The presented fracture surface was taken from the test specimens and was able to demonstrate a typical observation similar to the others. For the test specimen without the bolted clamp-up loaded under the stress range of 220 MPa, for example, the typical fracture surface and its magnification of approximately 30× under the scanning electron microscope (SEM TM3000, Hitachi, Tokyo, Japan) are shown in Figure 2a. On closer examination, the hole experienced crack initiation in the vicinity of the middle thickness of the steel plate. The crack initiation seemed to be moderately tied to the amount of disturbance at the hole surface. On the right side of the fracture surface, a straight primary crack initiated at the middle of the surface of the steel plate perpendicular to the upper surface of the plate, while the on the left side, some scattered cracks originated from the middle thickness point and propagated deeply into the plate. In addition, several tiny cracks can also be identified along with the primary cracks in the vicinity of the cavities, as shown in Figure 2a. In contrast, the specimen with the bolted clamp-up seems to exhibit typical crack initiation towards the upper edge of the hole, as shown in Figure 2b. This difference can be attributed to the preload of the bolt applied onto the upper and lower faces of the bolt clearance hole. In this sense, the fatigue crack propagation under tension stress is more likely to take place close to the clearance hole.

### 3.2. Stiffness Degradation Behaviour and Damage Progress

Stiffness is a well-defined engineering property, easily measured, and not involved in the destruction of the test specimen [23]. During the fatigue loading, damage accumulates with the crack propagation, which in turn progressively reduces the stiffness of the test specimens. The stiffness degradation behaviour is influenced by the connection details of the specimens. The stiffness degradation characteristics resulting from the fatigue damage can be supposed to be related with the damage propagation of the number of cycles. The stiffness at the *i*th cycle can be obtained as the ratio between the measured force range and the deformation range, which can be given by:

(1) $$R_{i}=\frac{\left(\sigma_{i,\text{max}}-\sigma_{i,\text{min}}\right)A_{m}}{\delta_{i,\text{max}}-\delta_{i,\text{min}}},$$

where σ*_i_*_,max_ and σ*_i_*_,min_ are the maximum and minimum stress components at the *i*th cycle; d*_i_*_,max_ and d*_i_*_,min_ are the deformation components corresponding to σ*_i_*_,max_ and σ*_i_*_,min_, respectively, which were measured from the testing machine and while eliminating the initial error of deformation arising from the grip assembly; *A*_m_ is the plate net cross-section area excepting the hole.

**Figure 3:**
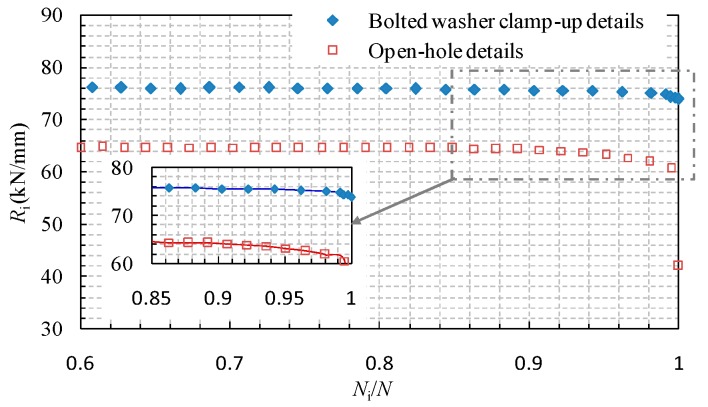
Comparison of stiffness degradation behaviour of specimens with open-hole details and bolted washer clamp-up details (Δσ = 300 MPa).

The comparison of the stiffness degradation behaviour of specimens with open-hole details and bolted washer clamp-up details is shown in Figure 3. It can be observed that the open-hole plate exhibits notable stiffness degradation when the nominal loading cycle is greater than 0.85. When approaching the ultimate state, the stiffness is decreased from approximately 65 kN/mm to 59 kN/mm. In contrast, the stiffness of the bolted clamp-up plate under similar loading force is nearly 76 kN/mm, which is 17% greater than that of the open-hole plate. Meanwhile, the nominal loading cycle for the initiation of the notable stiffness degradation is also enhanced to 0.9 and only a 2 kN/mm stiffness reduction until the ultimate loading case. Thus, it is apparent that the details of the bolted clamp-up condition significantly increase the initial stiffness and improve the stiffness degradation behaviour.

### 3.3. Fatigue Life Results and Analysis

**Figure 4:**
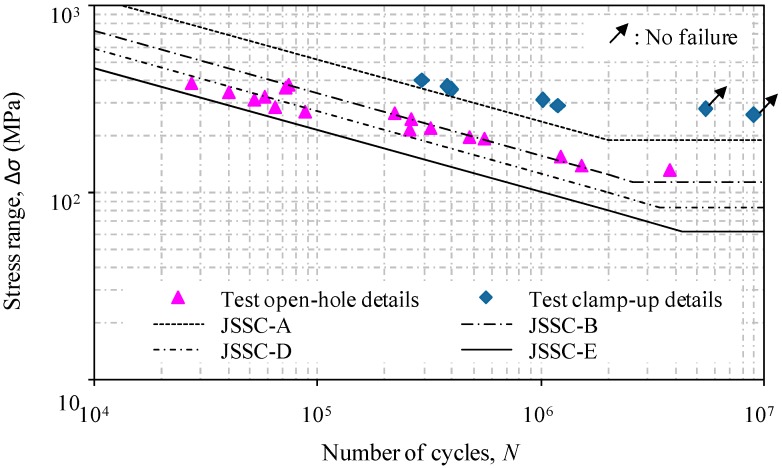
*S*-*N* relations of test specimens with open-hole details and bolted washer clamp-up details.

For each class of structural components, the general equation can be expressed in relating the number of cycles to failure, *N*, and the applied stress range, Δ*S*, as:

(2) $$N=C{\left(\Delta S\right)}^{-m},$$

where the exponent *m* is the slope of the *S*-*N* relation; *C* is the material constant-related parameter [23]. Taking the logarithm on both sides of Equation (2), the following equation can be written as:

(3) log(N)=log(C)−mlog(ΔS),

Following the general fatigue design rule, the test data can be processed based on a statistical analysis to provide the best fit mean *S*-*N* curve by the method of least squares. The fatigue detail categories of JSSC (Japanese Society of Steel Construction) [24] were also added for the purpose of comparison. A graphical presentation in the form of the stress range versus the number of cycles is shown in Figure 4 with codified detail classes.

Using regression analysis, the corresponding *S*-*N* relations with the curve slope equal to 3.0 for test specimens can be expressed as:

(i) For the specimens with open-hole details:
  (4) log(N)=12.45−3log(ΔS),
(ii) For the specimens with bolted washer clamp-up details:
  (5) log(N)=13.36−3log(ΔS)

**Figure 5:**
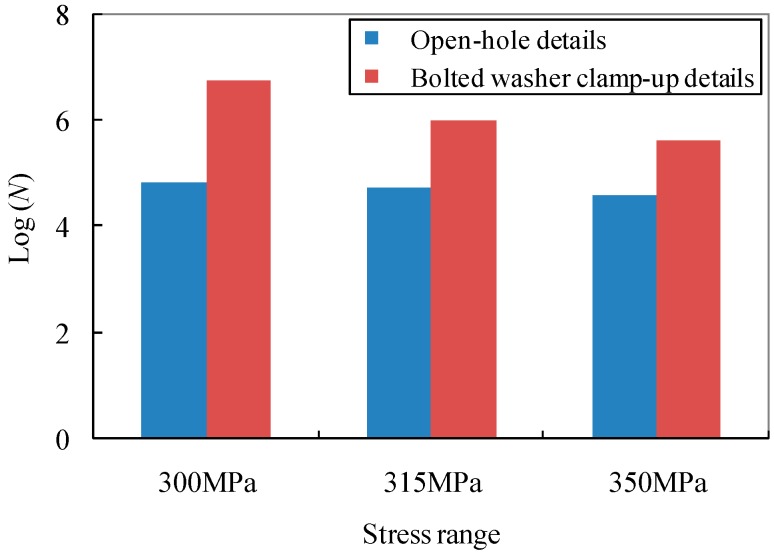
Comparison of fatigue life results of specimens with open-hole and bolted washer clamp-up details.

The comparison of the test data with assigned fatigue detail categories by calculating the given constant Log(*C*) of JSSC [24], AASHTO [6,25] and Eurocode 3 [26] is also listed in Table 2. It can be seen that the JSSC detail category *E* with a constant amplitude fatigue limit (CAFL) of 80, the Eurocode 3 detail category 100 with a constant amplitude fatigue limit (CAFL) of 100, and the AASHTO detail category *B*’ with a constant amplitude fatigue limit (CAFL) of 82.7 can be referred to as a lower bound in the evaluation of the test specimens with open-hole details. In contrast, the consideration of the bolted washer clamp-up is effective in the improvement of the fatigue life which can be referred to by JSSC detail category *A*, AASHTO detail category *A* and Eurocode 3 detail 160 with constant amplitude fatigue limits (CAFL) of 190, 165 and 160, respectively. For illustration, the evidence of the significant enhancement of the fatigue life of test specimens using the bolted clamp-up details can be observed in Figure 5.

## 4. Discussion

The fatigue behaviour of the open-hole steel plates differs when the clamp-up condition and varied hole fabrication methods are involved. Punching uses standard turret tooling to make parts which inevitably produce louvers, extrusions, tabs and some other shapes in addition to holes. As a result, the quality problems of tool marks and scratches are more prone to be present. Previous research work [17,18] showed that the failed punched specimen had a non-symmetrical fatigue crack which initiated near the transition zone between the cut and the tearing zones due to the punching process. In contrast, laser cutting as a non-contact process is ideal for parts that will be nested since the features requiring a forming operation such as louvers or extrusions are removed. Garcia et al. [19] indicated that the fracture surface of the laser-cut hole is featured by the fatigue cracks starting in the cut surface, favoured by the roughened surface. In this research, a similar observation can be obtained; however, the pattern of the fatigue crack is varied, as shown in Figure 2, within the range of the plate thickness and around the edge of the hole. This can be explained as the standard laser-cut hole being featured by the presence of predominant draglines within the thickness, which may trigger the fatigue cracks not only near the surface but also at the middle thickness point.

**Figure 6:**
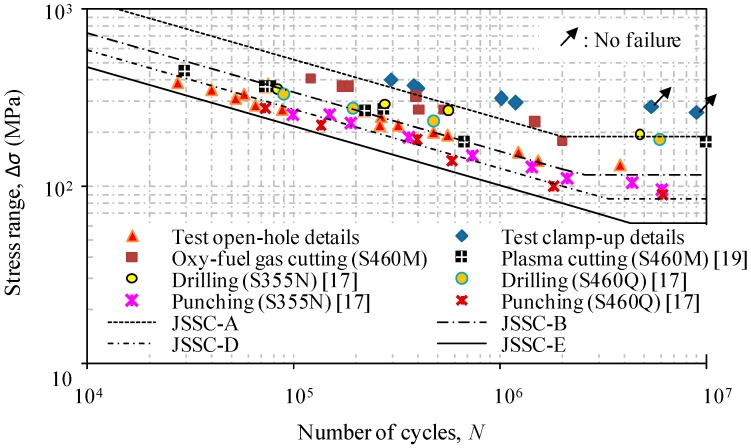
Comparison of fatigue life results of test specimens with reference using different hole fabrication methods.

**Figure 7:**
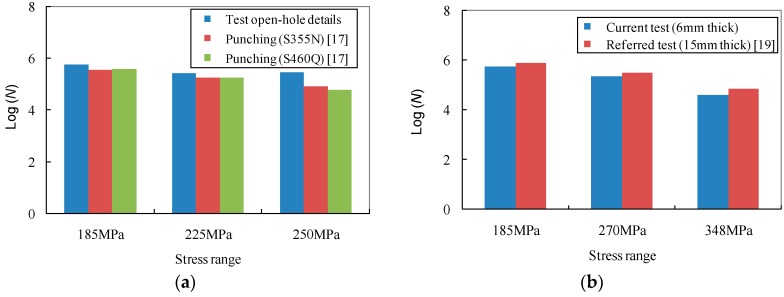
Comparison of fatigue life data influenced by details. (**a**) Influence of laser-cut and punched holes; (**b**) Influence of plate thickness.

For the sake of further discussing the influence of the clamp-up and hole fabrication methods on the fatigue life of the open-hole plates, the fatigue data of test specimens and the referred specimens of similar materials are summarized in Figure 6. Following the discussion in the foregoing paragraph, the fatigue life in the logarithm of the test results and the referred punched plates are compared in Figure 7a. It can be observed that the specimens with laser-cut holes exhibit 3%~10% greater fatigue life in the logarithm than those with punched holes under the stress ranges of 185 MPa, 225 MPa and 250 MPa. Likewise, this observation can be due to the removal of the contact problem induced by the punched hole which in turn extends the fatigue life to some extent. Therefore, the better fatigue behaviour of laser-cut holes as compared to punched holes can be confirmed and can be further considered for industrial application since the laser is also able to run large irregular cut-outs with much faster speed. On the other hand, the influence of plate thickness on the fatigue life of the test specimens can be identified from a comparison with referred test data [19]. As shown in Figure 7b, it can be seen that with the increase of the plate thickness from 6 to 15 mm, the test fatigue life in the logarithm of the test specimens is moderately enhanced by approximately 6%.

To compare the test fatigue life results with the referred specimens using other fabrication methods, the detail fatigue rating method–based analysis was adopted. The fatigue detail coefficient can be taken as the measure of the fatigue quality of a structural component [27]. Related coefficients resulting from this method indicate the inherent characteristics of the fatigue capacity of a structure independent of the applied fatigue load. The characteristic value of the Weibull distribution can be given as the relation between the number of cycles to failure, *N*, the number of test specimens, *n*, and the Weibull scale parameter, *m*, which is taken as 4.0:

(6) $$\beta ={\left[\frac{1}{n}\sum_{i=1}^{n}N_{i}^{m}\right]}^{\left(\frac{1}{m}\right)},$$

Assuming the required reliability, *R_s_*, is defined as 95%, the reliability level factor, *S_R_*, is defined as:

(7) $$S_{R}={\left[\ln\left(\frac{1}{R_{S}}\right)\right]}^{\left(\frac{1}{m}\right)},$$

**Figure 8:**
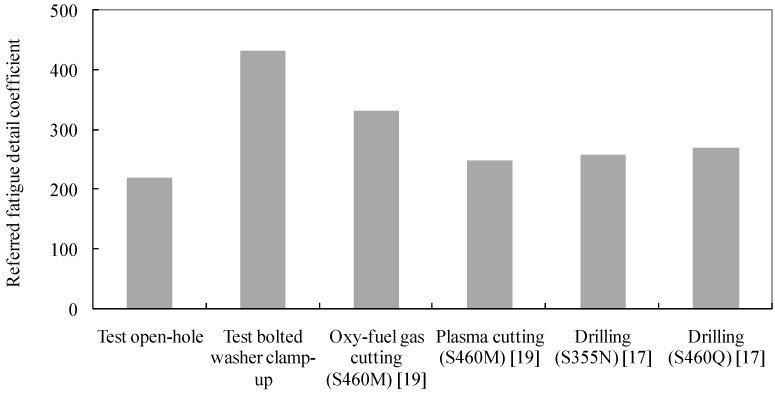
Fatigue detail coefficient of details with bolted clamp-up and varied cutting methods.

The fatigue life with a 95% level of reliability and a 95% confidence level for structural fatigue details is calculatedas: *N*_95/95_ = β/(*S*_R_*S*_c_). *S*_c_ is the confidence coefficient under the 95% confidence level [27]. Given the basic stress range level *N*_0_ at 50,000, the referred fatigue detail coefficient can be defined as σ_max_[*N*_95/95_/*N*_0_]^(1/*m*)^. Thus, σ_max_ = 290 MPa was considered for comparison. Using the above-mentioned method, the fatigue life results of the test specimens are compared with the reference regarding other fabrication methods as shown in Figure 8. It is demonstrated that the fatigue detail coefficients of the test open-hole specimens are 7%, 12% and 42% lower than that of the referred data with plasma cutting, drilling and oxy-fuel gas cutting, respectively, which can be due to the inherent shortcomings of the heat affecting zone induced by the laser cutting. In fact, the fatigue cracks are more prone to occur when these heat affecting zones are under a certain magnitude of tension load. On the other hand, the introduction of bolt washer clamp-up details significantly covers such shortages by enhancing the fatigue detail coefficient by 43%, 39% and 23% for the referred details with plasma cutting, drilling and oxy-fuel gas cutting, respectively. This can be attributed to the local compression applied by the bolt preload which lessens the surface tension crack initiation to some extent, and typical cracks are more likely to appear very close to the edge of the bolt clearance hole. In addition, the fatigue life of the structural components with laser-cut holes could be improved with the rational configuration of bolt clamp-up details.

## 5. Conclusions

The fatigue property of open-hole steel plates was investigated in this research with a focus on the influences of the bolted clamp-up condition and hole fabrication methods. Through analyzing the fracture surface, stiffness degradation and fatigue life results, the following conclusions can be drawn:

- The fatigue crack of test specimens mostly initiated not only at the edge of the hole but also in the vicinity of the middle thickness of the steel plate with the presence of predominant draglines within the thickness for the laser-cut hole.
- The open-hole plate using laser cutting exhibits notable stiffness degradation in its appearing cycle and magnitude. This trend seems to be reduced to some extent when the bolted clamp-up details are introduced.
- The bolted clamp-up details greatly enhance the fatigue life of the open-hole plate from category E to approaching category A as codified in JSSC, from category B’ to approaching category A as codified in AASHTO, and from category 100 to approaching category 160 as codified in Eurocode.
- The fatigue life of open-hole details with laser cutting is greater than that with punched holes but lower than those with plasma cutting, drilling and oxy-fuel gas cutting. The introduction of bolted clamp-up details can be used to compensate for the laser cutting holes when a lower fatigue detail coefficient is concerned.

Based on the basic understandings obtained from this study, further research is still needed incorporating a wider range of parameters, such as plate thickness, etc., in a follow-up study.

## Figures and Tables

**Table 1:** Chemical composition and mechanical properties of steel materials.

Steel Grade	Chemical Composition (%)					Mechanical Properties		
	C	Si	Mn	P	S	σ_y_ (MPa)	*E*_s_ (MPa)	σ_u_ (MPa)
Q345B	0.17	0.25	1.15	0.015	0.014	388	2.1 × 10^5^	553
S355N [17]	0.17	0.30	1.35	0.02	0.011	423	2.0 × 10^5^	602
S460Q [17]	0.08	0.30	1.31	0.014	0.003	619	2.0 × 10^5^	685
S460M [19]	0.12	0.45	1.49	0.012	0.001	484	2.05 × 10^5^	594

**Table 2:** Comparison of *S*-*N* relations with referred fatigue categories.

Categories/Relations	Standard Deviation	Log(*C*)	CAFL (MPa)
JSSC-*A*	-	13.13	190
JSSC-*B*	-	12.87	155
JSSC-*D*	-	12.30	100
JSSC-*E*	-	12.01	80
AASHTO-*A*	-	12.91	165
AASHTO-*B*	-	12.59	110
AASHTO-*B*’	-	12.30	82.7
Eurocode 3-160	-	12.91	160
Eurocode 3-112	-	12.45	112
Eurocode 3-100	-	12.30	100
Equation (4)	0.11	12.45	112.11
Equation (5)	0.06	13.36	225.22
